# Bacterial Signaling at the Intestinal Epithelial Interface in Inflammation and Cancer

**DOI:** 10.3389/fimmu.2017.01927

**Published:** 2018-01-05

**Authors:** Olivia I. Coleman, Dirk Haller

**Affiliations:** ^1^Technical University of Munich, Munich, Germany; ^2^ZIEL—Institute for Food & Health, Technical University of Munich, Munich, Germany

**Keywords:** intestinal epithelium, intestinal microbiota, bacterial signaling, inflammation, colorectal cancer

## Abstract

The gastrointestinal (GI) tract provides a compartmentalized interface with an enormous repertoire of immune and metabolic activities, where the multicellular structure of the mucosa has acquired mechanisms to sense luminal factors, such as nutrients, microbes, and a variety of host-derived and microbial metabolites. The GI tract is colonized by a complex ecosystem of microorganisms, which have developed a highly coevolved relationship with the host’s cellular and immune system. Intestinal epithelial pattern recognition receptors (PRRs) substantially contribute to tissue homeostasis and immune surveillance. The role of bacteria-derived signals in intestinal epithelial homeostasis and repair has been addressed in mouse models deficient in PRRs and signaling adaptors. While critical for host physiology and the fortification of barrier function, the intestinal microbiota poses a considerable health challenge. Accumulating evidence indicates that dysbiosis is associated with the pathogenesis of numerous GI tract diseases, including inflammatory bowel diseases (IBD) and colorectal cancer (CRC). Aberrant signal integration at the epithelial cell level contributes to such diseases. An increased understanding of bacterial-specific structure recognition and signaling mechanisms at the intestinal epithelial interface is of great importance in the translation to future treatment strategies. In this review, we summarize the growing understanding of the regulation and function of the intestinal epithelial barrier, and discuss microbial signaling in the dynamic host–microbe mutualism in both health and disease.

## Introduction

The human gastrointestinal (GI) tract represents the most densely colonized organ of the body, with the highest microbial load of 10^11^ bacteria/mL content in the colon (1, 2). Bacteria dominate the microbial ecosystem in the GI tract, with more than 90% belonging to the phyla *Bacteroidetes* and the *Firmicutes* (3–5). Despite considerable progress the functional complexity of the microbiome is still unresolved, and to date, mechanisms of microbe–host interactions involve a pleiotropic network of immune, metabolic, and trophic functions (1, 6). Studies in germ-free animals recognized the essential role played by the intestinal microbiome in the development and regulation of the mucosal immune system during early life (7–12). While many organisms have been shown to fulfill protective functions in the GI tract and are critical for host physiology, complex shifts in the community structure and abundance of certain microbes have been associated with the onset of inflammatory and tumorigenic diseases, such as inflammatory bowel diseases (IBD) and colorectal cancer (CRC) (6, 13–15).

Loss of epithelial barrier function and innate immunity are fundamental to the pathogenesis of inflammatory and infectious diseases. The intestinal immune system has the challenge of responding to pathogens, while remaining tolerant to food antigens and the commensal microbiota. The intestinal epithelium executes a compartmentalization between the lumen and the host, simultaneously acting as a selectively permeable first line of defense to fulfill its function of absorption, while maintaining an effective barrier against the intestinal microbiota, antigens and toxins. Intestinal epithelial cells (IECs) express pro-inflammatory cytokines in response to infectious invasive bacteria (16), but largely ignore non-pathogenic commensals (17). Certain intestinal pathogens (18, 19) and opportunistic commensals (20), however, can evade this first line of defense and enter IECs, suggesting that the existence of epithelial cell-intrinsic immune mechanisms for bacterial detection and limitation are essential. One key cell-autonomous mechanism of antibacterial defense is intestinal epithelial autophagy, shown to be activated following bacterial invasion through adaptor protein myeloid differentiation primary response gene-88 (MyD88) cell-intrinsic signaling, with autophagy-deficiency in mice causing increased dissemination of invasive bacteria (21), indicating that autophagy could have a broader role in inflammatory disease. IECs and innate immune cells of the lamina propria are able to differentiate self from non-self through a selective spatial and cellular expression of pattern-recognition receptors (PRRs) (22). Classically the detection of pathogen-associated molecular patterns (PAMPs) allows the intestinal epithelium to activate signaling pathways that induce the early host response to infection. The role of microbe-associated molecular patterns (MAMPs) in mediating innate recognition of the commensal “non-infectious” microbiota remains controversial. Paradoxically, recent progress in understanding IBD pathogenesis suggests that a defective innate immune system predisposes the host toward chronic inflammation (23, 24), supporting a protective role of PRR signaling in maintaining intestinal tissue homeostasis. Early work related to the activation of inflammation-related transcription factors, such as the nuclear factor kB (NF-kB), suggested a hormetic adaptation of the epithelium in response to commensal bacteria (25, 26), with elegant studies related to epithelial cell-specific inhibition of NF-kB activation validating the importance of this signaling pathway in maintaining tissue homeostasis (27). This paradigm shift was supported by Medzhitov and colleagues, demonstrating that microbiota-derived signals *via* the toll-like receptor (TLR)-related adaptor protein MyD88 protect mice from the development of colitis (28) and intestinal tumor formation (29). Thus, bacteria (dead or alive) and their metabolites form key mediators for the cross-talk between IECs and other mucosal cell types, through the interaction with host PRRs.

Although it is recognized that the intestinal microbiota has profound influences on health and disease, the understanding of the precise mechanism(s) by which this is exerted remains largely unknown (30). This review summarizes our knowledge of specific bacterial interactions and signaling mechanisms at the intestinal epithelial interface. We discuss bacterial signaling in inflammation and cancer, and reflect on how increasing knowledge of such mechanisms might be translated to the benefit of patient care.

## The Intestinal Epithelium: Our Dynamic Protective Barrier

**Figure 1:**
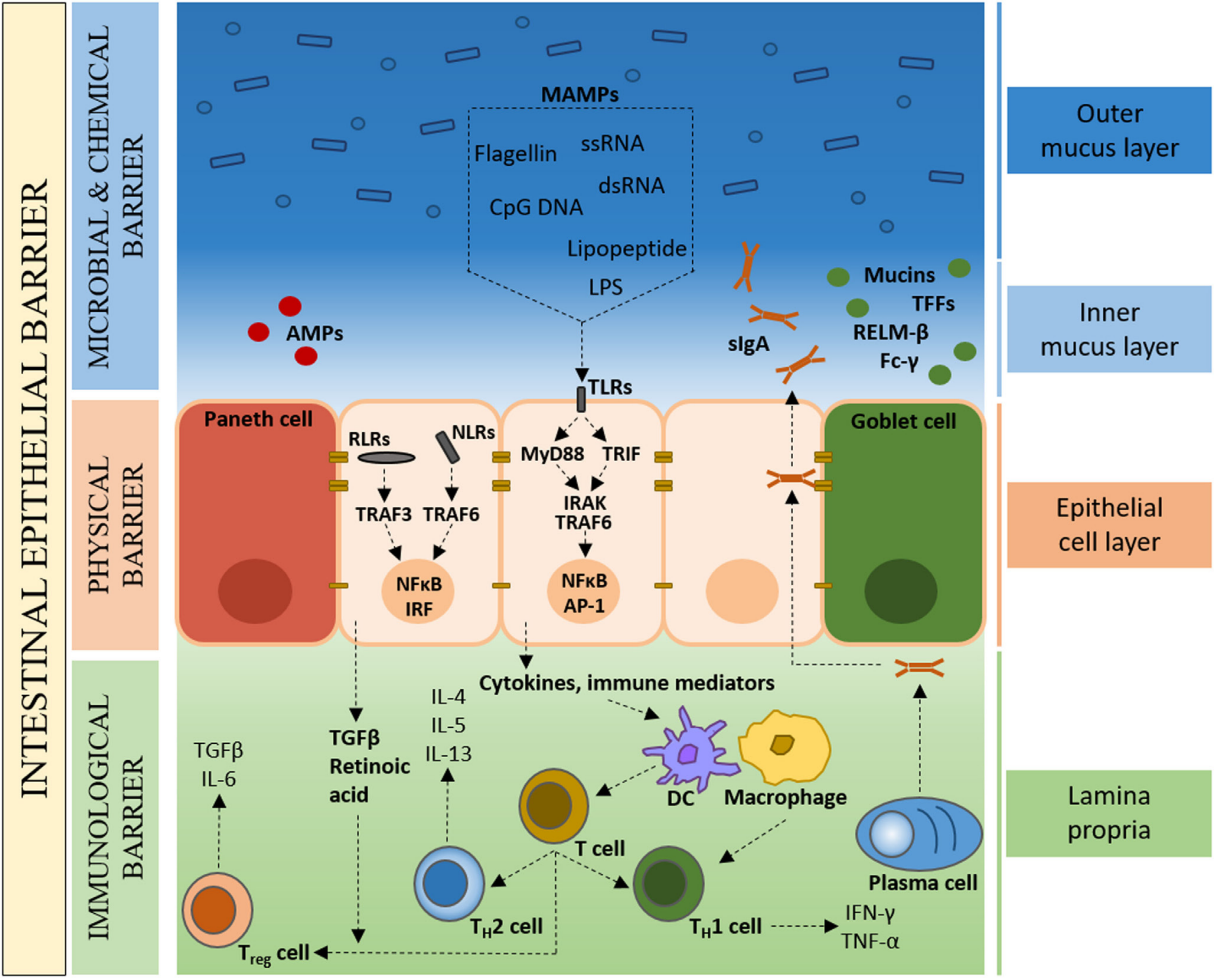
The colonic intestinal epithelium as a dynamic protective barrier. The single-cell layer (10 µm) of intestinal epithelial cells (IECs), which is comprised of distinct subpopulations, separates the luminal intestinal microbiota from the underlying tissue, forming a physical barrier. Overlying the IECs is the microbial and chemical barrier, mainly composed of the mucus layer(s). Goblet cells secrete mucins, which form a proteoglycan gel to create an inner mucus layer that is devoid of bacteria, and an outer mucus layer that forms a habitat for the intestinal microbiota. The largely sterile inner mucus layer has a high concentration of secretory immunoglobulin A (sIgA), antimicrobial peptides (AMPs), microbe-associated molecular patterns (MAMPs), as well as other bioactive molecules such as trefoil factor peptides (TFFs), resistin-like molecule β (RELMβ), and Fc-γ binding protein. Underlying the IECs, the Lipopeptide/lipoprotein (LP) contains mainly plasma cells, macrophages, and dendritic cells that, in the healthy state, are of a naïve nature with limited expression of inflammatory cytokines.

In spite of the symbiotic nature of the microbe–host relationship, the close proximity of bacteria to intestinal tissue poses a considerable health challenge. An effective and dynamic intestinal epithelial barrier is therefore crucial to conserve a compartmentalized microbe–host interaction and tissue homeostasis (Figure 1). In the healthy organ, the epithelium maintains a distinct anatomical barrier relevant for a constant state of homeostasis, while being exposed to a myriad of environmental stimuli that include, but are not limited to, microbes, dietary products and inorganic materials (31). A single-cell layer of IECs forms a continuous physical barrier, with tight junctions connecting adjacent IECs and associating with cytoplasmic actin and myosin networks that regulate intestinal permeability (32). Long-lived pluripotent stem cells located at the base of intestinal crypts continuously produce tissue-specific precursor cells that transit through a differentiation pathway that gives rise to absorptive lineage cells (enterocyte/colonocyte) or secretory lineage cells (goblet, Paneth, enteroendocrine and tuft) (33). IECs represent not only a physical barrier but also contribute to intestinal health through the production of mucus (goblet cells) and the secretion of antimicrobial peptides (AMPs) (Paneth cells).

Goblet cells secrete mucin glycoproteins, of which Muc2 is the main constituent of the approximately 150-µm thick (in the mouse) colonic mucus layer (34). While the mucus layer in the small intestine consist of a single layer, in the colon, two structurally distinct mucus layers are formed; an inner mucus layer that is devoid of bacteria, and an outer mucus layer that forms a habitat for a large number of bacteria (35, 36). In addition to mucins, goblet cells secrete a range of bioactive molecules such as trefoil factor peptides (TFFs), resistin-like molecule β (RELMβ), and Fc-γ binding protein (37). Intestinal Paneth cells are the main source of AMPs that function in host defense and in establishing and maintaining the intestinal microbiota (38, 39). Secretory immunoglobulin A (sIgA) directed against intestinal bacteria and produced by Lipopeptide/lipoprotein (LP) plasma cells, binds the polymeric immunoglobulin receptor (pIgR), and transcytoses across the epithelium to prevent microbial translocation across the epithelial barrier (40, 41). This concerted interplay between plasma cells and IECs provides an adaptive immune element to the intestinal epithelial barrier. Also found scattered throughout the LP are T cells, stromal cells, and antigen presenting cells such as dendritic cells (DCs) and macrophages. Specialized IECs, called microfold (M) cells, and goblet cells facilitate the transport of luminal antigens and bacteria across the intestinal epithelial barrier to DCs, with macrophages sampling through trans-epithelial dendrites (42–44). Under steady-state conditions, the intestinal immune system detects commensal bacteria and provides basal signals without the full activation of adaptive immune responses (7).

The intestinal microbiota forms part of the intestinal barrier by limiting bacterial colonization and stimulating epithelial turnover (45). For example, *Bifidobacteria* species produce high concentrations of the short-chain fatty acid (SCFA) acetate, and can thereby prevent enteropathogenic *Escherichia coli* (EHEC) infection and its Shiga toxin release (46). Similarly, butyrate-producing *Fecalibacterium prausnitzii, Eubacterium rectale*, and *Roseburia* species directly target virulence gene expression to prevent bacterial infection (47). Studies have demonstrated that bacteria-dependent signals regulate the intestinal epithelial barrier and contribute to its effective functioning. Experiments in germ-free mice have shown that mucus layer thickness is reduced compared with conventionally housed mice, and that stimulation with lipopolysaccharide (LPS) and peptidoglycan (PGN) can reverse this to SPF-like levels of mucus thickness (48). Similarly, AMP and antimicrobial protein production, transcriptional- and post-translational regulation can be dependent on and enhanced by the presence of intestinal microbial signals (49–51). TLR, NOD-like receptor (NLR), RIG-like receptor (RLR), and C-type lectin receptor (CLR) family members provide distinct microbial signaling pathways in the intestinal epithelium (52–57). Despite evidence from mouse models deficient in PRRs and signaling adaptors (27, 52–56, 58), there is further need for epithelial-specific PRR knock-out mice to fully comprehend the role of bacteria-derived signals in intestinal epithelial homeostasis and repair.

## Bacterial Recognition at the Intestinal Epithelial Interface

Of the four signaling receptor families (TLR, NLR, RLR, and CLR), members of the TLR family of type I transmembrane proteins are the best-characterized receptors in the intestinal mucosa. NLRs are cytoplasmic receptors, of which nucleotide-binding oligomerization domain-containing protein 1 (NOD1) and NOD2 functions have been well characterized, that signal to elicit cytokine, chemokine, and defensing expression (59). RLRs recognize viral RNAs and induce innate antiviral responses (60). TLRs can be located at the cell surface or internal cell compartments, respond to specific ligands, and are associated with particular adaptors that activate downstream signaling cascades. Nearly all TLRs are expressed in the human colon, with the expression of TLR1, TLR2, TLR3, TLR4, TLR5, and TLR9 demonstrated in IECs of the human small intestine (61). Studies have identified four main adaptor molecules [MyD88, MyD88-adapter-like (Mal/TIRAP), TIR domain-containing adaptor-inducing interferon-β (TRIF), and TRIF-related adaptor molecule (TRAM)] that interact with TLRs in response to ligand stimulation (62, 63).

With the exception of TLR3, all TLRs signal *via* the adaptor protein MyD88, whose engagement triggers signaling cascades that ultimately lead to the activation of transcription factors such as NF-ĸB, interferon regulatory factor (IRF) and activator protein 1 (AP-1) (64). While the lack of MyD88 in certain mouse strains was shown to have a significant impact on the composition of the intestinal microbiota, linking TLR signaling to the structure of the microbial community (65), a study published the same year using MyD88- and TLR-deficient mice and wild-type littermates, demonstrates that colony and housing differences between laboratories make it difficult to clearly define the influence of innate immune signaling pathways on the microbiota (66). Here, Ubeda et al. found that MyD88 and TLR signaling does not detectably alter the composition of the intestinal microbiota, demonstrating the need for caution in the interpretation of microbiota analysis in mutant mice. It is important to bear in mind that observations in MyD88-deficiency do not imply a direct link to microbial signals, but may in fact be intrinsic. Besides TLR receptors, MyD88 associates with all receptors of the IL-1 cytokine family, and contributes to tissue homeostasis, including tissue repair and regeneration (28, 67, 68). Therefore, the inability of MyD88-deficient mice to respond to the IL-1 cytokine family is likely also involved. In the colon epithelium, for example, it was shown that the protective effect of MyD88 is, at least in part, mediated by the IL-1 cytokine family member IL-18 (69).

The monoassociation of germ-free mice with the prominent gut commensal *Bacteroides fragilis* revealed that this bacterium specifically signals through TLR2 on regulatory T cell *via* its polysaccharide A (PSA) symbiosis factor, to enable its niche-specific mucosal colonization (70). Similarly, the colonization of mice with *B. fragilis* protects against experimental colitis in a TLR2-dependent manner (70, 71). Monocolonization in germ-free rats with the commensal *Bifidobacterium lactis* was shown to cause TLR2-mediated MAPK and NF-ĸB pathway activation in IECs (72). Furthermore, The colonization of germ-free rodents with *Enterococcus faecalis* or *Bacteroides vulgatus* activate NF-ĸB signaling and induce chemokine expression in colonic IECs through TLR2 and TLR4 signaling, respectively (26, 73).

A study in TLR5-deficient mice showed that the cecal microbiota differed from wild-type littermates in >100 bacterial phylotypes (74), indicating that TLR signaling has implications in the regulation of the intestinal microbiota. This was also shown in MyD88-deficient mice that demonstrated higher levels cecal Rikenellaceae and Porphyromonadaceae families (75). In the healthy state, mice deficient in TLR signaling (MyD88-deficient, TLR4-deficient, MyD88/TRIF-knockouts) do not show any differences in proliferation and IEC barrier function compared with wild-type mice (76, 77). Under conditions of injury, however, MyD88-, TLR2-, and TLR4-deficient mice show increased susceptibility to dextran sodium sulfate (DSS)-induced colitis (28, 77, 78). Despite the importance of PRRs in the bidirectional crosstalk between the intestinal microbiota and the host, studies in PRR-deficient mice have shown that only those deficient in TLR5, NLRP6, or RIG-I develop spontaneous intestinal inflammation (79–81). This may suggest a major role of compensatory mechanisms, where PAMPs are recognized by multiple synergizing host PRRs that share key innate immune signaling pathways, resulting in a sufficient host response to commensal bacteria in PRR-deficient mice that do not show spontaneous phenotypes. It is important to consider that not all laboratories and animal colonies observe spontaneous basal inflammation in the above-mentioned PRR-deficient mice (82).

## Bacterial Signaling Mechanisms in Intestinal Inflammation

Despite difficulties in assigning the intestinal microbiota to the role of cause or consequence, chronic mucosal and, in particular, GI inflammation is linked to an imbalance of commensal bacteria and their gene products in patient groups with IBD (83–87). IBD is the collective name for multifactorial chronic relapsing inflammatory infections of the intestinal tract, which primarily includes Crohn’s disease (CD) and ulcerative colitis (UC). IBD can be debilitating and may lead to life-threatening complications. The development of IBD is characterized by a change in the normal intestinal microbiota (dysbiosis), with a reduction in both bacterial quantity and bacterial diversity (83, 88–90). In the context of IBD, microbiota analyses have negatively associated *Faecalibacterium prausnitzii* and *Akkermansia municiphila* with the disease, whereas *Escherichia coli, Fusobacterium nucleatum, Haemophilus parainfluenzae, Veillonella parvula, Eikenella corrodens*, and *Gemella moribillum* were shown to be positively associated with the inflammatory disease (86, 91–94). Dysbiosis is associated with a breakdown of host–microbial mutualism, with accumulating evidence from numerous scientific disciplines firmly implicating such a breakdown in mutualism in the pathogenesis of IBD (95, 96).

Abnormal PRR signaling is implicated in the development of chronic intestinal inflammation. The cytosolic NLR NOD2 (also known as CARD15) recognizes bacterial PGN-derived muramyl peptide (MDP) to elicit NF-ĸB-mediated proinflammatory responses and AMP synthesis (97–99). *Nod2*-deficient mice harbor an elevated load of commensal resident bacteria, display dysbiosis, and show a reduced ability to prevent intestinal pathogen colonization (100, 101). In turn, NOD2 expression is dependent on the intestinal microbiota, suggesting a feedback mechanism in the maintenance of intestinal homeostasis (101). In line with the above findings, Nod2 gene mutations were identified in patients with CD (102, 103), suggesting that Nod2 gene mutations may be associated with changes in the commensal microbiota that may facilitate disease progression.

The genetically engineered interleukin-10-deficient mouse (IL-10^−/−^) provides a model of spontaneous intestinal inflammation (104) and has been extensively used as an experimental tool to mirror the multifactorial nature of IBD. Evidence for the requirement of resident enteric bacteria for the development of colitis in IL10^−/−^ mice stemmed from studies in germ-free animals, where colitis development was not observed (105). It has been shown that the gram-positive intestinal bacterium *E. faecalis* drives distal colonic inflammation in IL-10^−/−^ mice following monoassociation (106, 107). Furthermore, increased mucosal growth of, and specific antibody-titers against, *E. faecalis* have been shown in patients with UC, also correlating with disease severity (108, 109). Findings from our own group identified that the virulence factor gelatinase E (GelE) partially impairs intestinal epithelial barrier integrity in IL-10^−/−^ mice (110), and that the colitogenic activity of *E. faecalis* was partially and almost completely abrogated when deficient for the enterococcal polysaccharide antigen (Δ*epaB*) and lipoproteins (Δ*lgt*) envelope structures, respectively (111). Monoassociation of IL-10^−/−^ mice with the commensal bacteria *E. faecalis, E. coli*, or *Pseudomonas fluorescens* demonstrated that different commensal species selectively initiate distinct immune-mediated intestinal inflammation in the same host (107). Such results invite the hypothesis that particular microbial effectors, or a combination of effectors from different bacteria, are required to elicit pathogenesis or maintain the necessary barrier function for intestinal homeostasis. Additionally, not only the specific bacterium, but the susceptibility of the host plays a major role in disease progression, as shown by the induction of colitis by *Bacteroides vulgatus* in HLA/B27-β2m transgenic rats, but not in IL-2^−/−^ mice (107, 112, 113).

Identifying bacterial gene products that drive protective rather than pathogenic inflammation in the intestine is crucial to rebalance homeostasis in inflammatory diseases and malignancies. *Lactobacillus* species, such as *Lactobacillus acidophilus*, are normal inhabitants of the intestinal microbiota and have received considerable attention as beneficial ecosystem members (114, 115). Several studies have shown that TLR2 regulates epithelial barrier function and enhances tight junction formation, as well as playing a crucial role in driving acute intestinal inflammation, but not chronic intestinal inflammation (116–118). *L. acidophilus* stimulates DCs through TLR2 *via* lipoteichoic acid (LTA) to trigger the production of inflammatory and regulatory cytokines (119–121). Deletion of the phosphoglycerol transferase gene (LBA0447) that synthesizes LTA generated an *L. acidophilus* derivative (NCK2025) that diminishes colitis when administered orally in a murine colitis model (122), confirming the role of LTA in inducing inflammation (123, 124). Of note here is that LTA, among others, may not present a true TLR2 ligand, as the large number of structurally diverse putative ligands may rather show their effects due to lipopeptide/lipoprotein (LP) contamination. In another example, *L. paracasei*, a single strain derived from the VSL#3 bacterial mixture clinically shown to reduce inflammation in IBD patients (125–127), was found to secrete the prtP-encoded protease lactocepin with anti-inflammatory effects *via* the degradation of proinflammatory chemokines (128, 129).

Collectively, the above findings support the notion that the colitogenic activity of opportunistic pathogens can be assigned to specific bacterial structures, and that such characterizations are indispensable in understanding host–microbe interactions relevant for the development of intestinal inflammation.

## Bacterial Signaling Mechanisms in CRC

Colorectal cancer is one of the leading causes of death in the western society, being ranked third most lethal neoplasia in the United States in both men and women (130). Multiple lines of evidence show that the gut microbiota plays a major role in CRC development, both quantitatively and qualitatively. The significant role played by bacteria in inflammation-driven tumorigenesis is evident by the decreased tumor formation found in several CRC mouse models housed in germ-free conditions (131–133), or under antibiotic treatment (134). Accordingly, the inhibition of microbial recognition through the loss of PRR signaling or T-helper cell activation leads to a diminished neoplastic transformation (29, 131, 135, 136). Numerous bacterial species including, but not limited to, *Streptococcus bovis, Bacteroides fragilis*, and *E. coli* have been found in CRC samples. The best-known association is that of *S. bovis* bacteremia and CRC (137). It was demonstrated that *S. bovis* and its wall antigens induce IL-8 production, leading to the formation of nitric oxide (NO) and reactive oxygen species (ROS), which contribute to the neoplastic process (138). More recently, *Peptostreptococcus anaerobius* was identified as a candidate to be significantly enriched in the stool and mucosa of patients with CRC (139–141). A study assessed the association of *P. anaerobius* in stool and colonic tissue from patients with colorectal adenomas and adenocarcinomas, providing mechanistic insights that the actions of *P. anaerobius* are mediated *via* interaction with TLR2/4 on host cells to induce ROS production, increase cholesterol biosynthesis, and activate pro-oncogenic factors and pathways to promote CRC (142).

Approximately 80% of sporadic colorectal tumors are associated with mutations in the adenomatous polyposis coli (APC) gene (143); a central gatekeeper protein in CRC. Multiple intestinal neoplasia mice with a point mutation in *Apc* (*Apc*^Min^*^/+^*) mimic sporadic cancer and familial adenomatous polyposis, and have been used to study the role of TLR signaling in intestinal tumorigenesis through the crossing with MyD88-deficient mice (MYD88-deficient × *Apc*^Min^*^/+^*). While tumor incidence was similar in these mice compared with *Apc*^Min^*^/+^* mice, a reduction in tumor number and size was observed, which was linked to a reduced expression of the tumor growth-promotor COX2 (29, 144). These data suggest that TLR signaling is involved in tumor growth, but not tumor initiation. Further evidence for the contribution of TLR signaling to the development of sporadic cancer, and colitis-associated cancer, stemmed from the use of TLR4-deficient mice that were protected against tumorigenesis following azoxymethane (AOM) and DSS treatment (145). Furthermore, TLR4 activation on tumor cells can prevent their lysis, thereby protecting cancer cells (146). This is of particular relevance with regard to cancer treatment strategies, as the immunosuppressant drug Rapamycin decreases TLR4 expression and its prostaglandin E2 production (147). Findings from animal models of CRC are corroborated by human studies; the TLR4/MyD88 co-receptor complex showed enhanced expression in approximately 20% of CRC patient samples, compared with normal mucosae and adenomas (148, 149).

Mechanistically, bacteria may promote tumorigenesis by numerous processes, including toxic metabolite production and genotoxic biosynthesis (150), thereby providing further CRC treatment tactics. One study aimed at inhibiting toxic effects of colibactin toxin-producing *E. coli*; frequent colonizers of CRC (151). Here, two identified boronic acid-based com pounds were shown to bind to the active site of the ClbP enzyme involved in the synthesis of colibactin, and shown to suppress DNA damage and tumorigenesis induced by *pks*-harboring [conserved genomic island coding for nonribosomal peptide synthetases (NRPS) and polyketide synthetases (PKS) bacteria (152)]. These findings not only confirm the role of colibactin toxin-producing *E. coli* in carcinogenesis but also provide a novel family of inhibitors to target pks-harboring bacteria in the treatment of CRC.

Injection of specific bacteria into tumor tissue may help eradicate tumors through the stimulation of inflammation and anti-tumor responses (153). In line with the above comment that a combination of multiple effectors may be necessary to maintain homeostasis or elicit pathogenesis: two bacteria can be better than one in cancer immunotherapy. A recent study applied an approach to cancer immunotherapy through the use of an attenuated *Salmonella typhimurium* strain engineered to secrete *Vibrio vulnificus* Flagellin B (FlaB) (154). Zheng et al. showed that FlaB-mediated tumor suppression is associated with TLR5-mediated host reactions and dependent on TLR4 and MyD88 signaling, as shown with TLR/MyD88 knockout mice and cell lines. Evidently it is feasible that non-virulent tumor-targeting bacteria can release multiple TLR ligands, and can be used as cancer immunotherapeutics.

In a latest study, Sahu et al. linked the dysbiotic behavior of a constitutively invasive variant of commensal non-pathogenic *E. coli* to CRC tumorigenesis (155). Aberrant host invasion leads to realignment of multiple host signal transduction cascades through reciprocal modulation of microbe sensing pathways Nod1/Rip2 and TLR/MyD88, leading to an expansion of the cancer stem cell population. This supports the notion that microbe-driven tumorigenesis may result from self-derived and contextual cues, which determine the role of such microbes in homeostasis and carcinogenesis, rather than strict correlations with commensal virulence.

*Lactobacillus acidophilus* NCK2025, discussed earlier with regards to the regulation of inflammation in a colitis model, was investigated in a mouse model of colonic polyposis (TS4Cre × APC^lox468^) to assess its moderation of pathogenic inflammation within the tumor microenvironment (156). Khazaie et al. reported that oral treatment with the LTA-deficient *L. acidophilus* NCK2025 normalized innate and adaptive pathogenic immune responses, causing a regression of established precancerous colonic polyps. This work demonstrates the ability of the probiotic strain with anti-inflammatory properties to reverse preneoplasia, rendering this *L. acidophilus* strain as a potential candidate for regulating intestinal immunity in the protection against inflammation and CRC susceptibility. Additional investigations are key to further characterize bacterial gene products that can influence inflammation to restore intestinal homeostasis, to provide novel avenues for the treatment and prevention of inflammatory and cancer pathologies.

In light of high-sensitivity-detection of pre-cancerous lesions still posing a great challenge, the potential of fecal microbiota for the early-stage detection of CRC was recently investigated (157). In a metagenomic sequencing study to identify taxonomic markers in CRC patients, Zeller et al. found functional and taxonomic associations with CRC from noninvasive fecal sample readouts. Furthermore, general dysbiosis common to inflammation was addressed by including published metagenomes from IBD patients in the marker species classifier, showing that stronger associations were observed with CRC, with only modest influences by inflammation-related microbiota changes. This study demonstrates the possibility of CRC detection from fecal microbial markers, and the potential for further identification of cancer-associated differences in gene function, gene content and genomic variation through additional metagenomic data.

## Concluding Remarks

**Figure 2:**
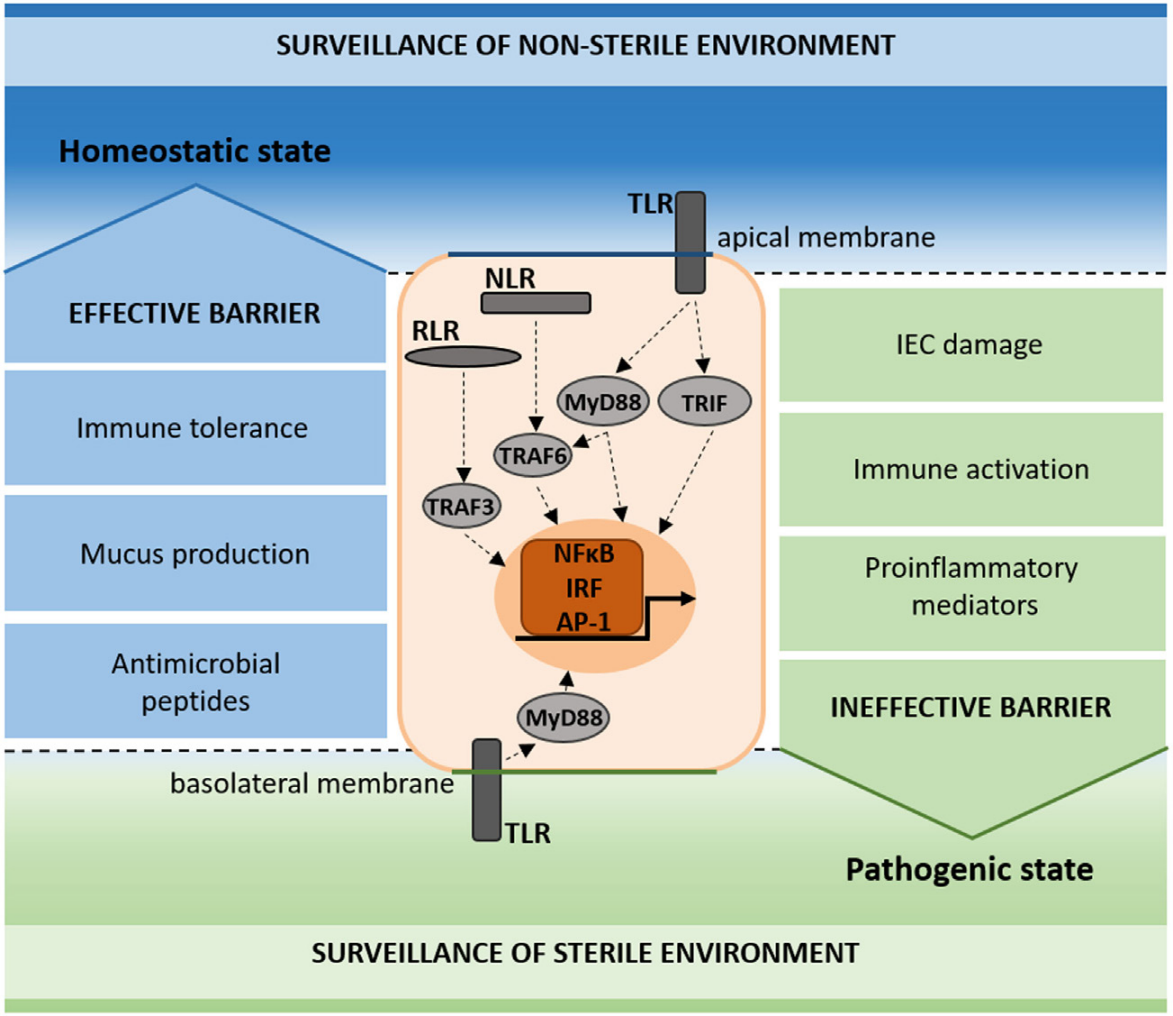
Schematic representation of pattern recognition receptor (PRR) surveillance in the homeostatic and pathogenic state. PRRs (TLR, NLR, and RLR) signal on the apical and basolateral membrane of intestinal epithelial cells (IECs), contributing to the surveillance of the non-sterile (apical) and sterile (basal) environments. In the homeostatic state, immune tolerance, mucus production, and antimicrobial peptides add to the maintenance of an effective barrier (blue). In the pathogenic state, IEC damage, immune activation, and proinflammatory mediators result in an ineffective barrier (green).

Over the years, it has become evident that the intestinal microbiota is not merely a bystander in the complex events that regulate intestinal homeostasis, but that it plays a fundamental role in eliciting both beneficial and detrimental effects in the host. Collectively, the studies outlined in this review highlight the diverse and multifaceted roles of IECs and the intestinal microbiota in the maintenance of intestinal homeostasis, and the complexity of the relationship between the two. The diverse barrier functions of the intestinal epithelium play a crucial role in microbe–host mutualism. Cells of the intestinal epithelium express a range of PRRs that sense and respond to a variety of microbial signals to maintain an effective barrier and respond to pathogens (Figure 2). Evidence of the importance of PRR signaling stems from studies in mice with specific defects in such signaling pathways, which show increased susceptibility to developing disease (28). Regarding the host side of the mutualism, future studies to increase our understanding of how mucus, AMPs, and sIgA dynamics can be regulated to maintain barrier function will provide avenues to develop therapeutic interventions for preserving intestinal homeostasis. Probiotic and prebiotic treatment options available to consumers are currently drawn from a narrow range of organisms. Increasing knowledge of the intestinal microbiota with its constituents is changing this paradigm; however, due to the complex and dynamic nature of the intestinal ecosystem, the mechanistic understanding of the integration of bacterial signals remains a great challenge to this field. Antibiotics selectively targeting bacterial pathogens have been extensively used in the prevention and treatment of numerous diseases (158, 159). In light of antibiotics disrupting the composition of the enteric intestinal microbiota and promoting antibiotic resistance, future mechanistic experimental efforts to elucidate (yet) unidentified mechanisms of bacterial effector proteins to enable the development of novel drugs aimed at targeting rather than killing bacterial pathogens, seems like the logical step forward. To this end, animal models of inflammation and cancer provide useful approaches to demonstrate functionality, given the high interindividual variation and nature of studies using human cohorts.

## Author Contributions

All authors listed have made a substantial, direct, and intellectual contribution to the work and approved it for publication.

## Conflict of Interest Statement

The authors declare that the research was conducted in the absence of any commercial or financial relationships that could be construed as a potential conflict of interest. The reviewer MH declared a past coauthorship with one of the authors, DH, to the handling editor.
